# Increased Health Risk in Subjects with High Self-Reported Seasonality

**DOI:** 10.1371/journal.pone.0009498

**Published:** 2010-03-03

**Authors:** Nicolas M. Øyane, Reidun Ursin, Ståle Pallesen, Fred Holsten, Bjørn Bjorvatn

**Affiliations:** 1 Department of Public Health and Primary Health Care, University of Bergen, Bergen, Norway; 2 Department of Biomedicine, University of Bergen, Bergen, Norway; 3 Department of Psychosocial Science, University of Bergen, Bergen, Norway; 4 Department of Psychiatry, University of Bergen, Bergen, Norway; 5 Norwegian Competence Center for Sleep Disorders, Haukeland University Hospital, Bergen, Norway; University of Otago, New Zealand

## Abstract

**Background:**

Seasonal variations in mood and behaviour, termed seasonality, are commonly reported in the general population. As a part of a large cross-sectional health survey in Hordaland, Norway, we investigated the relationship between seasonality, objective health measurements and health behaviours.

**Methodology/Principal Findings:**

A total of 11,545 subjects between 40–44 years old participated, completing the Global Seasonality Score, measuring seasonality. Waist/hip circumference, BMI and blood pressure were measured, and blood samples were analyzed for total cholesterol, HDL cholesterol, triglycerides and glucose. Subjects also completed a questionnaire on miscellaneous health behaviours (exercise, smoking, alcohol consumption). Hierarchical linear regression analyses were used to investigate associations between seasonality and objective health measurements, while binary logistic regression was used for analysing associations between seasonality and health behaviours. Analyses were adjusted for sociodemographic factors, month of questionnaire completion and sleep duration. Seasonality was positively associated with high waist-hip-ratio, BMI, triglyceride levels, and in men high total cholesterol. Seasonality was negatively associated with HDL cholesterol. In women seasonality was negatively associated with prevalence of exercise and positively associated with daily cigarette smoking.

**Conclusions/Significance:**

High seasonality was associated with objective health risk factors and in women also with health behaviours associated with an increased risk for cardiovascular disease.

## Introduction

Variations in mood and behaviour across the seasons, also known as seasonality, are reported by most humans in the Western countries to a certain degree [1]–[4]. Seasonal Affective Disorder (SAD) is characterized by major depressive episodes occurring exclusively during a particular time of the year [5], and could be the result of a high seasonality combined with a high vulnerability for depression [6], [7]. In contrast to the classical vegetative symptoms linked with depression, SAD-patients typically report hyperphagia, weight gain and increased sleepiness during depressive episodes. From an evolutionary point of view, high seasonality has been proposed to represent a phylogenetic advantage, as it could result in energy storage and promote reproductive potentials by coinciding with seasons optimal for conception, gestation and lactation [8]. However, in the modern society, with access to plentiful resources year round, seasonality may conversely be a stress factor. Recently we reported associations between high levels of seasonality, low socioeconomic status and depression [4], [9].

A study from the general population in the US reported the average weight gain between fall and spring to be 0.48 kg in a normal population, and the weight gain was not reversed by the next fall [10]. Since subjects with high seasonality tend to gain more weight during winter months, the risk for obesity could presumably be increased over the years. A recent study by Rintamaki and colleagues reported waist circumference and prevalence of the metabolic syndrome to increase with increasing seasonality in Finland [11]. Post-hoc analyses did not reveal any significant associations between serum lipid levels and seasonality. However, Pjrek et al. recently reported total cholesterol levels to be lower in SAD-subjects than in non-seasonally depressed or schizophrenic patients [12]. No differences between these patient groups were found for triglyceride levels. Conversely, Morgan et al found low plasma cholesterol to be associated with symptoms of non-seasonal depression [13]. Other authors report the opposite [14] or no relationship at all [15]. Associations have also been found between non-seasonal depression and high triglyceride levels [16], although that finding was not replicated in a study including men only [17].

Associations between seasonality and health behaviours have previously not been reported. Both smoking, sedentarism and high alcohol consumption have been reported to be associated with cardiovascular disease [18]–[21], and it is therefore interesting to investigate these health behaviours in subjects with high seasonality.

We investigated the impact of high seasonality on objective health measurements, blood parameters and subjective risk factors for cardiovascular disease. To our knowledge, this is the largest study on seasonality so far including both objective and subjective data, and we seek to answer the following questions:

1. Is high seasonality associated with objective risk factors for cardiovascular disease?
2. Is health behaviour significantly different in subjects with high vs. low seasonality score?

## Materials and Methods

### Ethics Statement

The study protocol was approved by Regional Committee for Medical and Health Research Ethics, West Norway (REK-West) and by the Norwegian Data Inspectorate. Written consent was given by the participants for their information to be stored in the database and used for research.

### Procedure

The cross-sectional Hordaland Health Study (HUSK) was conducted from 1997 to 1999 by the National Health Screening Service, the University of Bergen, and local health services. All individuals in Hordaland county, Norway, born between 1953 and 1957 (being 40–45 years old at the time of investigation) were invited by postal mail to participate in the study (n = 29 400). A total of 8 598 men and 9 983 women participated, yielding a participation rate of 63% (57% for men and 70% for women). Two out of five possible questionnaires were completed by the subjects. A general health questionnaire, including questions about health behaviours, was completed by all subjects before attending the screening station. At the screening station, the participant randomly received one of two gender-specific questionnaires to be filled in at home. For women, both questionnaires contained items concerning general degree of seasonal fluctuations in mood and behaviour (The Global Seasonality Score - GSS), whereas for men, the GSS items were included only in one of the two questionnaires due to space limitations. In sum, half of the men (randomly selected) and all women were offered the GSS questionnaire. Of these, 3432 men (79.8%) and 8113 women (81.2%) completed the questionnaire. The data collection lasted for one year, and the dates of questionnaire completion were noted by the staff receiving the questionnaire. Due to summer vacation among the staff, no subjects met at the screening station in July, and a relatively low number of subjects met at the screening station in August. The data in the HUSK study are available for all participating research groups, and other research groups may apply to access the data.

### Global Seasonality Score (GSS)

The Global Seasonality Score (GSS) is a central subscale of the Seasonal Pattern Assessment Questionnaire (SPAQ), widely used in epidemiological studies of seasonality and SAD. Due to space requirements, the complete SPAQ was not included in HUSK. The GSS retrospectively measures seasonal variations by six different items (sleep, mood, weight, energy, social activity and appetite). Each of the six items of the GSS are scaled “0” for no seasonality, “1” for mild, “2” for moderate, “3” for marked and “4” for extremely marked change in seasonality, yielding a total score ranging between 0–24. Higher scores indicate higher degree of seasonal variation. The cut-off scores of the GSS are usually set to 8–10 for sub-SAD and ≥11 for SAD, and subjects also have to report distress due to experienced seasonality in order to get a SAD-diagnosis [2], [22]–[24]. In lack of better estimates, we defined the respondents in the current study as either having low (GSS <8, n = 7013), moderate (GSS 8–10, n = 2099) or high (GSS ≥11, n = 2433) seasonality. We used an authorized Norwegian version of the questionnaire, translated by Dr. Lingjærde (University of Oslo). The GSS has been shown to have an acceptable reliability and validity in epidemiological studies [25], [26].

### Objective Health Measurements

Subjects' height, weight, waist circumference and hip circumference were measured (shoes and outer garment were taken off) by study personnel. In addition, specially trained nurses measured blood pressure and collected blood samples. Systolic and diastolic blood pressures were measured in a relaxed sitting position following two minutes of rest. During the rest period, the participants were told that three automated blood pressure measurements were to be performed, and that no talking was allowed during these measurements. The average of the last two measurements was used as the recorded blood pressure. Blood samples were collected non-fasting, and later analyzed for total-cholesterol, HDL-cholesterol, triglycerides and glucose.

### Health Behaviours

All participants completed questionnaires with questions on various health behaviours. They were asked about the number of beers, glasses of wine and glasses of spirits consumed in a two-week period, and the number of days per month they consumed alcohol. A question concerning daily cigarette smoking (yes/no) was also included. Subjects were asked about weekly quantities of low intensity exercise (not resulting in perspiration or short breath) and high intensity exercise (resulting in perspiration and short breath), with the following four possible response categories; “0 hours”, “ less than 1 hour”, “1–2 hours”, “3 hours or more”. Both exercise variables were dichotomized into exercising at least 3 hour per week vs. exercising less than 3 hour per week. Subjects were also asked whether or not they used medications for hypertension. The questions regarding various health behaviors are not validated, but already used in numerous health surveys in Norway coordinated by the Norwegian Institute of Public Health.

### Statistics

The analyses of associations between seasonality and health measurements/behaviour were broken down by gender. Analyses of variance were conducted using seasonality group as the risk and predictor variable, while objective health measurements (waist-hip ratio, BMI, total cholesterol, HDL cholesterol, triglycerides, glucose, systolic/diastolic blood pressure) and health behaviours (low/high intensity exercise, alcohol consumption, cigarette smoking) comprised the dependent variables. The Kruskal-Wallis test was used for multiple comparisons of ordinal data (percentage of subjects exercising equal to or more than three hours per week and percentage of subjects being daily smokers) while a one-way Analysis of Variance (ANOVA) was used for the other parameters. Post-hoc analyses were performed using the Least Significant Difference (LSD) test. Hierarchical multiple linear regression analyses were performed to investigate associations between seasonality and objective health risk factors. Waist-hip-ratio, BMI, total cholesterol, HDL cholesterol, triglycerides, glucose, systolic blood pressure and diastolic blood pressure were used as dependent variables. Triglycerides values were inverted in order to obtain a normal distribution, as the original values were considerably skewed to the left. Two blocks of independent variables were entered. The first block included month of questionnaire completion, marital status, income, education, living area and sleep duration, while the second block consisted of the Global Seasonality Score. In addition, a binary logistic regression analysis was performed, using GSS (≥11 vs. <11) as an independent variable. Low and high intensity exercise (at least 3 hour/week vs. <3 hour/week), alcohol consumption (≥8 days per month vs. <8 days per month) and cigarette smoking (yes vs. no) were used as dependent variables. The analyses were adjusted for annual income, education, marital status, month of questionnaire completion, urban/rural residence and sleep duration. Sleep duration was inserted as a continuous variable, while the other adjusting variables were inserted as categorical variables. To investigate whether any associations were significantly affected by the season in which the subjects were examined, we ran an ANOVA inserting objective health measurements as the dependent variables, while seasonality group, season and the interaction term seasonality group*season comprised the independent variables. Additionally, we ran a logistic regression analysis using health behaviours as dependent variables and seasonality group, season and the interaction term seasonality group*season as independent variables. Finally, we constructed a figure depicting the prevalence of obesity (BMI>30), high waist-hip ratio (>1 for men or >.85 for women), hypertension (systolic blood pressure >140 mm Hg or diastolic blood pressure >90 mm Hg), hypertriglyceridemiae (triglycerides >2 mmol/l), high total cholesterol (total cholesterol >5 mmol/l) and low HDL-cholesterol (HDL-cholesterol <1 mmol/l) in the different seasonality groups. Missing data were excluded from the analyses, and partially complete responses were discarded. Imputation was not used. SPSS for Windows, version 15.0 was used for all the statistical analyses. A two-tailed p-value<.05 was chosen to indicate statistical significance.

## Results

### Multiple Comparison Analyses: Objective Health Measurements Factors and Health Behaviours in the Different GSS Groups (Table 1)

When looking at objective health measurements in men, weight, BMI, waist-hip ratio and triglycerides significantly increased with increasing GSS. Among health behaviours in men, high intensity exercise (at least 3 hours per week) was significantly less commonly reported in the moderate and high GSS groups (14% and 16%, respectively) than in the low GSS group (20%). Prevalence of daily cigarette smoking was positively associated with the GSS.

**Table 1:** Objective health measurements and health behaviours in different seasonality groups (n = 11,545).

Health parameter	Gender	GSS <8 (n = 7013)	GSS 8–10 (n = 2099)	GSS ≥11 (n = 2433)	Statistics		
					aKruskal Wallis X^2^(2) bANOVA F(2)	P	Post Hoc^c^
Waist-Hip ratio	Male	.90 (.06)	.91 (.06)	.91 (.06)	9.1^b^	<.001	2
	Female	.79 (.06)	.79 (.06)	.80 (.06)	11.9^b^	<.001	2,3
Body Mass Index	Male	25.9 (3.4)	26.2 (3.3)	27.1 (3.6)	27.5^b^	<.001	2,3
	Female	24.5 (40)	24.7 (39)	25.4 (43)	33.7^b^	<.001	2,3
Total Cholesterol (mmol/l)	Male	5.70 (1.0)	5.72 (1.1)	5.80 (1.1)	2.2^b^	ns	
	Female	5.38 (.95)	5.38 (.97)	5.43 (.96)	1.2^b^	ns	
HDL Cholesterol (mmol/l)	Male	1.12 (.28)	1.11 (.27)	1.09 (.27)	2.7^b^	ns	
	Female	1.41 (.37)	1.40 (.34)	1.37 (.32)	4.4^b^	.013	2
Triglycerides (mmol/l)	Male	2.11 (1.5)	2.20 (1.6)	2.31 (1.5)	4.6^b^	.010	2
	Female	1.31 (.79)	1.33 (.80)	1.46 (1.0)	13.1^b^	<.001	2,3
Glucose (mmol/l)	Male	5.3 (1.2)	5.2 (1.0)	5.4 (1.5)	1.9 b	ns	
	Female	5.1 (1.0)	5.1 (1.0)	5.1 (1.0)	1.9 b	ns	
Systolic blood pressure (mm Hg)	Male	131 (14)	131 (13)	131 (15)	0.2^b^	ns	
	Female	123 (14)	123 (14)	123 (14)	1.1^b^	ns	
Diastolic blood pressure (mm Hg)	Male	76 (10)	77 (9)	77 (11)	0.4^b^	ns	
	Female	71 (10)	71 (10)	71 (10)	0.2^b^	ns	
Low intensity exercise (at least 3 h per week) (%)	Male	44 (50)	41 (49)	38 (49)	5.6^a^	ns	
	Female	47 (50)	45 (50)	41 (49)	16.5^a^	<.001	1,3
High intensity exercise (at least 3 h per week) (%)	Male	20 (40)	14 (35)	16 (37)	14.2^a^	.001	
	Female	11 (31)	10 (30)	10 (29)	3.2^a^	ns	
Alcohol consumption (days per month)	Male	4.0 (4.2)	4.1 (4.0)	4.1 (4.4)	0.3^b^	ns	
	Female	2.6 (3.4)	2.9 (3.4)	2.7 (3.1)	2.9^b^	ns	
Cigarette smoking (%)	Male	32 (47)	35 (48)	37 (48)	6.6^a^	.04	2
	Female	31 (46)	37 (48)	44 (50)	87.2^a^	<.001	1,2,3

Means are compared by using ANOVA for normally distributed data and Kruskal-Wallis for non-normally distributed data. Standard Deviations are shown in parentheses.

GSS  =  Global Seasonality Score.

a Kruskal-Wallis statistics.

b ANOVA statistics.

c Post-Hoc analysis (using Least Squares Difference and 0.05 significance level) were reported as follows: 1- Significant difference between the GSS <8 and GSS 8–10 groups, 2- Significant difference between the GSS <8 and GSS ≥11 groups and 3- Significant difference between the GSS 8–10 and GSS ≥11 groups.

In women, weight, BMI, waist-hip ratio and triglycerides were positively associated with GSS, while HDL-cholesterol was negatively associated with GSS. Among health measurements in women, prevalence of low intensity exercise (at least 3 hours per week) was significantly lower in the high GSS group (41%) compared with the moderate and low GSS groups (47% and 45%, respectively). Prevalence of daily cigarette smoking were positively associated with GSS (Table 1).

### Associations between Seasonality and Objective Health Measurements (Figure 1, Tables 2 and 3)

**Figure 1:**
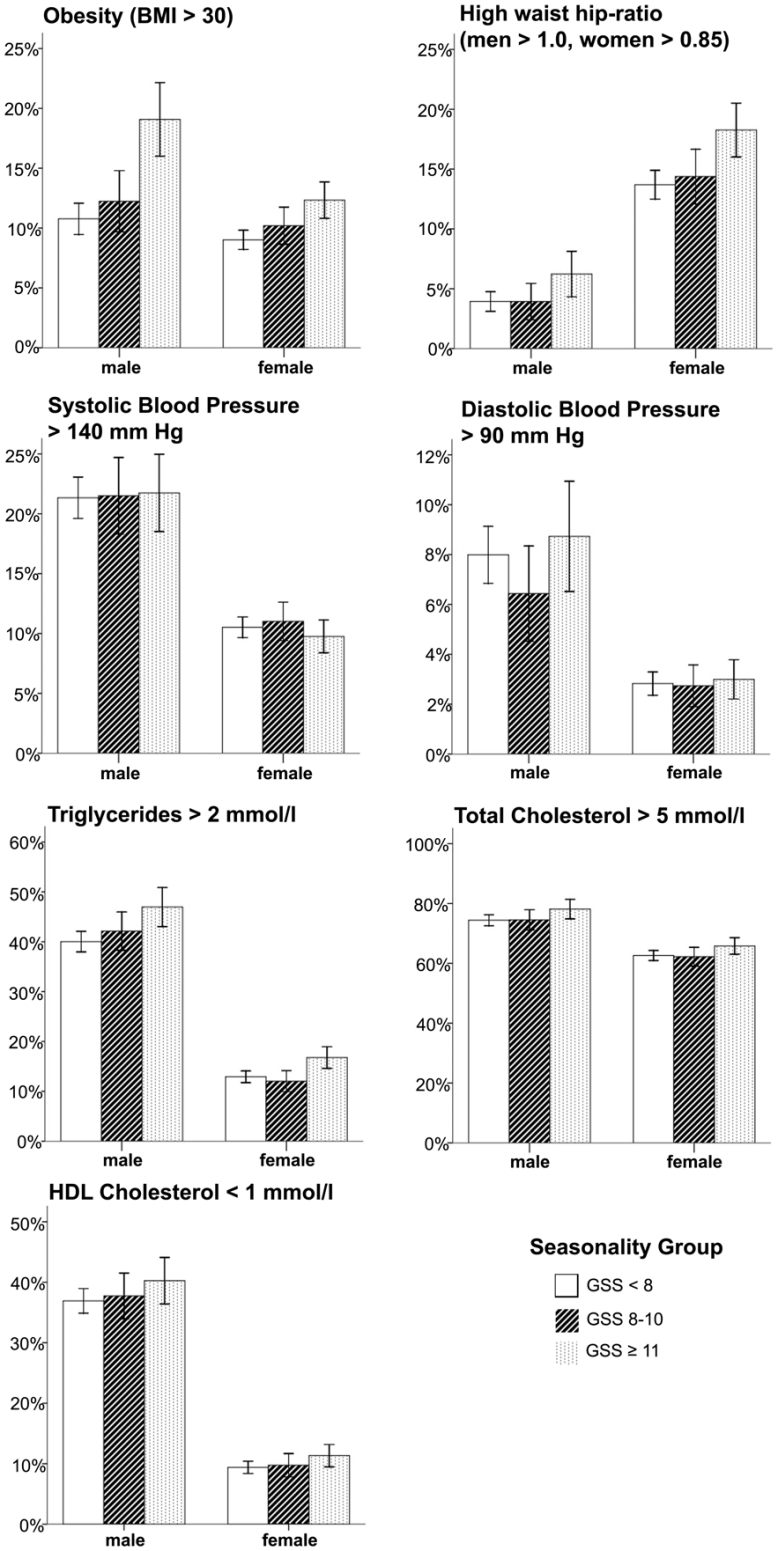
Prevalence of objective health risk factors associated with cardiovascular disease in different seasonality groups. Each chart has prevalence of health risks on the vertical axis and seasonality group on the horizontal axis, and results for men and women are shown separately. GSS = Global Seasonality Score. Vertical lines depict the 95% Confidence intervals.

Increasing GSS was significantly associated with increasing waist-hip ratio, BMI, triglycerides and decreasing HDL-cholesterol in both men and women (Figure 1, Table 2). In men, increasing GSS was also significantly associated with increasing total cholesterol. When looking at season of investigation, the interaction term seasonality group*season was significantly associated with systolic blood pressure in both gender and BMI in women (table 3). Systolic blood pressure was lower during summer in subjects reporting high seasonality compared with subjects reporting low seasonality. In women, BMI varied more across seasons in subjects with high seasonality than in subjects with low seasonality. However, BMI was highest in women with high seasonality than in women with low seasonality regardless of season.

**Table 2:** The impact of the Global Seasonality Score on objective health risk factors.

	Global Seasonality Score			
	Men		Women	
	β	t	β	t
**Waist-Hip ratio**	.066	3.70***	.058	3.75***
**BMI**	.115	6.40***	.079	5.13***
**Total Cholesterol**	.040	2.22*	.015	NS
**HDL Cholesterol**	−.039	−2.14*	−.031	−2.01*
**1/Triglycerides**	−.070	−3.87***	−.069	−4.46***
**Glucose**	.006	NS	.014	NS
**Systolic Blood pressure**	−.001	NS	−.025	NS
**Diastolic Blood pressure**	.027	NS	.000	NS

Hierarchic linear regression model controlled for month of questionnaire completion, marital status, income, education, living area and sleep duration (n = 11,544).

β: Standardized regression coefficient; NS: not significant.

*p<.05;

***p<.001.

**Table 3:** Effect of season on the associations between high seasonality and objective health risk factors.

Health measurement	Seasonality group	Season				Statistics for seasonality group*season	
		Winter	Spring	Summer	Fall	Anova F(6)	P
Men							
Systolic blood pressure (mm Hg)	GSS<8	132 (13)	131 (14)	130 (13)	131 (15)	2.60	.016
	GSS 8–10	133 (13)	130 (13)	129 (14)	132 (13)		
	GSS≥11	134 (15)	131 (12)	123 (20)	129 (17)		
Women							
BMI	GSS<8	24.6 (4.0)	24.6 (4.1)	24.2 (3.7)	24.3 (3.8)	2.34	.030
	GSS 8–10	24.4 (3.8)	24.7 (3.8)	25.0 (4.1)	24.8 (4.1)		
	GSS≥11	25.5 (4.5)	25.3 (4.2)	24.8 (3.8)	25.6 (4.4)		
Systolic blood pressure (mm Hg)	GSS<8	124 (14)	123 (14)	122 (15)	123 (14)	2.21	.039
	GSS 8–10	123 (13)	123 (13)	123 (14)	123 (14)		
	GSS≥11	123 (12)	124 (15)	119 (12)	123 (13)		

Analysis of variance using objective health measurements as dependent variables and seasonality group, season and the interaction term seasonality group*season as independent variables. Only significant effects are shown.

**GSS = Global Seasonality Score.**

### Associations between High Seasonality and Health Behaviours (Table 4)

Seasonality was significantly and negatively associated with low and high intensity exercise and positively associated with daily cigarette smoking in women. In men, no significant associations were found between high seasonality and health behaviours. When looking at season of investigation, the interaction term seasonality group*season was not significantly associated with any of the health behaviours (Table 4).

**Table 4:** The impact of High seasonality on health behaviours.

HEALTH PARAMETER	Men		Women	
	Odds-ratio	95% CI	Odds-ratio	95% CI
**Low intensity exercise (at least 3 hours per week)**	0.92	0.76–1.11	0.80	0.68–0.94**
**High intensity exercise (at least 3 hours per week)**	0.91	0.71–1.18	0.75	0.57–0.99*
**High alcohol consumption (≥8 days per month)**	0.97	0.75–1.27	1.06	0.82–1.37
**Daily cigarette smoking (%)**	1.10	0.90–1.35	1.42	1.20–1.67**

Logistic regression analysis using Global Seasonality Score (GSS) as the predictor variable and objective health risk factors/health behaviours as criterion variables (n = 11,544). The analyses are adjusted for annual income, education, marital status, month of completing the questionnaire, urban/rural residence and sleep duration.

CI: Confidence Interval.

*P<0.05.

**P<0.01.

## Discussion

Seasonality was significantly and positively associated with objective health risk factors: Waist-hip ratio, BMI, triglycerides, and negatively with HDL cholesterol. In men seasonality was significantly associated with total cholesterol level. On the other hand, we did not find a higher prevalence of hypertension or use of antihypertensive drugs in our subjects with high seasonality. Additionally, we found exercise to be less common and daily cigarette smoking to be more common among women with high seasonality than in women with moderate or low seasonality. The association between seasonality and BMI in women was affected by the season of measurements; still BMI was higher in women with high seasonality than in women with low seasonality regardless of season.

Overall it seems to be fair to conclude that subjects with high seasonality have an elevated risk for cardiovascular disease. In a population-based study as the present one, it is important to distinguish between clinical and statistical significance of our findings. Since seasonality is common in the general population, even small effect sizes might represent considerable public health consequences including health economical concerns. From an epidemiological point of view, results from cross-sectional surveys are best suited to generate hypotheses. Longitudinal studies on the other hand, are more suitable to test such hypotheses. The main reason for this is that the direction of causality is impossible to determine in any cross-sectional study, and unknown or uncontrollable confounding factors could therefore affect the results.

Seasonality can lead to increasing psychological distress, which is known to be associated with unhealthy behaviour [27]. Sher et al. (2001) suggested that SAD could suppress the immune system and affect the risk of having cardiovascular disease during winter [28], and this could also be the case with high seasonality per se. Furthermore, low levels of outdoor light exposure during winter months might cause inadequate resetting of the circadian clock, which presumably is linked to obesity [29]. On the other hand, high seasonality might be a result of and not necessarily a cause of objective health risk factors. For instance, obese subjects could find seasonal changes to be more distressing than other subjects. Moreover, some chronic diseases (such as asthma and rheumatoid disease) worsen in cold weather during winter months in Norway, which in turn could increase immobility and obesity [30], [31].

The theoretical significance of the link between blood lipid levels and mood disorders is unclear. We here report a significant positive association between triglyceride levels and seasonality. This is in accordance with a previous study, where patients suffering from SAD had higher triglyceride levels than controls [12]. Another study reported higher serum triglyceride levels in patients with non-seasonal depression than in control subjects [16]. Studies on depressed patients have reported lower [13], [32] or higher [14] serum cholesterol levels in depressed patients compared to control subjects. In the study by Pjrek et al. [12], serum cholesterol levels were reported to be significantly lower in SAD-subjects compared with other subjects, unlike our results. Further studies are needed to investigate whether it is seasonality per se or the associated behavioural changes which are linked with higher triglyceride levels.

### Strengths and Limitations

This is the first large health survey investigating associations between seasonality, objective health measurements and health behaviours. One of the major strengths of this study is the fact that it was based on a larger health survey, diminishing the potential risk of selection bias. Many previous studies in this field used specific patient populations, which tend to over-estimate the prevalence of comorbidity due to help-seeking behaviour [33]. Having emphasized this, it is important to bear in mind that this study is not investigating SAD, but seasonality in general. We did not investigate to which extent subjects regarded seasonality to be problematic in their daily life. Hence, the classification of our population into different seasonality groups could be argued to be of uncertain clinical validity. However, the GSS itself is a reliable questionnaire, and the cut-off values used in SAD studies are the best approximated values we could find for grouping subjects. As the number of women was much larger than the number of men in this study, the statistical power in the analyses for women was considerably higher than for men, as illustrated by the larger confidence intervals for men. There were a moderate number of non-responders in this study, possibly affecting our results. Non-responders usually report more psychopathology than the rest of the population [34]. In a similarly designed study from another part of Norway, non-responders were reported to have poorer health and a higher mortality rate [35], [36]. Hence, the subjects in the present study probably represent a relatively healthy part of the population of Hordaland. The subjective data in the present study were based upon self-report, and any significant association could reflect an underlying disposition of negative affectivity, which in turn could lead to an overestimation of the true association between different self-reported outcomes [37]. Moreover, only subjects aged 40–44 years were invited to participate in the study. SAD prevalence is reported to be lower in this age group compared with younger subjects [38], while the prevalence of risk factors for cardiovascular disease rises with age [39]. This could potentially affect our results. The blood samples were taken non-fasting. Since glucose, triglyceride levels and, to some extent, total cholesterol are affected by recent food intake, this weakens the accuracy and precision of these measurements. Consequently, confidence intervals broaden and the chance of finding significant associations with seasonality consequently decreases.

### Conclusions

In this first large study on the association between seasonality, objective health measurements and health behaviours, we found high seasonality to be significantly associated with various objective health risk factors. In women, high seasonality was also associated with health behaviours associated with an increased risk for cardiovascular disease. Limited by our cross-sectional design, the reported relationships could reflect that a high degree of seasonality is associated with an increased risk for vascular disease and thus unfavourable for humans in the modern society.
